# Albumin 20% in surgical and critically ill patients; a comprehensive review

**DOI:** 10.1186/s13054-026-05959-1

**Published:** 2026-03-24

**Authors:** Robert G. Hahn, François Jardot, Randal O. Dull, Joachim Zdolsek, Patrick Y. Wuethrich

**Affiliations:** 1 Karolinska Institutet at Danderyds Hospital (KIDS), Stockholm, 171 77 Sweden; 2 Department of Anaesthesiology and Pain Medicine, Inselspital, Bern University Hospital, University of Bern, Bern, Switzerland; 3 Departments of Anesthesiology, Pathology, Physiology, College of Medicine, University of Arizona, Tucson, AZ USA; 4 Department of Anesthesiology and Intensive Care, Department of Medical and Health Sciences, Linköping University, Linköping, Sweden

**Keywords:** Hyper-oncotic albumin, Plasma expansion, Fluid balance

## Abstract

**Background:**

Hyper-oncotic (20%) albumin has long been used during surgery and intensive care, but its pharmacology and relevant clinical use have not been adequately summarized.

**Main body:**

Hyper-oncotic albumin expands the plasma volume by twice the infused volume due to colloid pressure-induced recuitment of interstitial fluid, which primarily occurs via the lymphatic route. The interstitium and also the whole body is dehydrated, which helps maintaining the intravascular space well filled also at low arterial pressures. Albumin reduces the capillary permeability by volume exclusion and by transporting sphingosine-1-phosphate to the endothelial surface. The plasma volume expansion has a half-life of 6–10 h. During surgery, albumin 20% provides long-term plasma volume expansion, counteracts edema, and is effective in compensating hemorrhage. Its most apparent use is for de-escalation in intensive care, which involves positive effects on microcirculation and lung function. The mortality might be reduced in septic shock, which needs final validation. Our group has identified six situations in which albumin 20% can be expected, or has shown to be, of clinical value. These are: (1) Intraoperatively where large amounts of crystalloid threaten to cause adverse effects. (2) For plasma volume expansion in patients with peripheral edema, in particular if the urine output is low. (3) Gastrointestinal surgery with major hemorrhage where intestinal suturing is needed. (4) De-escalation in the ICU. (5) Septic shock. (6) Lung injury including pleural effusions and respiratory distress.

**Conclusion:**

Hyper-oncotic albumin effectively increases the plasma volume, dehydrates the interstitium, and improves the microcirculation. These characteristics can be beneficial to patients in selected clinical situations.

## History of use

Albumin-containing saline solutions have an 80-year long history as an infusion fluid in clinical medicine. Settings where such fluids have an established role include liver disease, nephrotic syndrome, and pediatrics. Less certain benefits are offered in general surgery and intensive care. Here, albumin has been given to increase the plasma volume, to correct hypoalbuminemia, and to achieve a scavenger effect. However, its use was questioned after publication of a widely cited meta-analysis 1998 by the Cochrane group, reporting increased mortality in patients receiving albumin [1]. The fear of increasing the mortality has subsided but is replaced by a concern that colloids in general harm the kidneys. Specifically, reports of renal injury related to the use of hydroxyethyl starch in septic patients [2] resulted in a virtual collapse of colloid use in general surgery [3]. However, gradually more albumin seems to be prescribed in surgical intensive care [4] despite clinical recommendations that advocate crystalloid fluid for plasma volume expansion (PVE) in settings where colloids have not proved to be superior. At present, there are virtually no reports of improved long-term benefits [5].

The present report reviews the clinical pharmacology and use of 20% albumin, which is special due to its high oncotic pressure. Knowledge about how 20% albumin behaves in the body can help the clinician to identify situations when this fluid might offer benefits. The use of 20% albumin and selected studies with iso-oncotic (4–5%) albumin during surgery and in ICU settings are reviewed. The effects of albumin 20% are likely to be transferable to albumin 25%, which is the formulation available in the United States. Special areas of use, such as pediatrics and liver diseases, will not be discussed.

## Properties of albumin

### Qualitative benefits

Albumin is the dominant protein in human plasma and responsible for 70–80% of its colloid osmotic pressure (COP). Albumin consists of a negatively charged polypeptide chain of 585 amino acids and a mass of 66 kDa. It is synthesized in the liver in response to the interstitial COP and has a half-life in the body of 2–3 weeks. Albumin serves as a carrier for several endogenous and exogenous compounds, as a scavenger of reactive oxygen and nitrogen species, and operates as a buffer molecule for the acid–base equilibrium [6]. The plasma concentration of albumin per se is relevant for the pharmacokinetics of drugs, since many drugs and hormones bind to albumin [7]. These properties have not yet been used in clinical strategies.

Most of the clinical benefits of albumin 20% that were identified in a review by Jacob et al. in 2008 are attributable to a powerful plasma volume expanding (PVE) effect [8]. The cardiac output response to volume loading is stronger compared to crystalloid fluids after cardiac bypass surgery [9] and there is less edema in the gastrointestinal wall [10]. Hyper-oncotic albumin is also more effective than saline in maintaining the arterial pressure during hemodialysis [11].

Other effects are due to the increase in plasma COP that occurs when infusing albumin 20%. Disability was reduced after brain injury due to less pronounced brain edema after combining albumin 20% with furosemide administration [12]. However, this study might be at odd with the SALT sub-study that reported higher mortality in 460 patients with brain injury who were resuscitated with the iso-oncotic albumin (5%) as compared to saline (33.2% vs. 20.4%) [13], which Myburgh extrapolated to 20% albumin [14].

Later studies generally support these early conclusions by Jacob et al. [8]. Hence, albumin 20% has a greater fluid- and sodium-sparing effect than albumin 5% in the ICU patients, which means that less volume can be given to achieve the same PVE [15, 16]. The hemodynamics is better preserved with colloids than with crystalloids in both volunteers [17] in ICU patients [18]. In cardiac surgery, the interstitial space becomes less volume expanded using albumin [19] and smaller amounts of vasopressors are needed postoperatively compared to crystalloids [20]. Crystalloid fluid 30 mL/kg elicited signs of pulmonary edema in volunteers, which was not seen after infusing 6 mL/kg of 20% albumin [17].

The concerns about the safety of colloids that arose in 2012 due to adverse effects of hydroxyethyl starch cannot be extrapolated to albumin. Compared to Ringer´s, albumin 20% does not increase the incidence of kidney injury in septic patients [21]. Moreover, albumin carries the lowest risk of inducing dilutional coagulopathy among all available colloid fluids [22]. Hence, Rasmussen et al. reported that, unlike hydroxyethyl starch [23], albumin 5% did not increase the blood loss during major surgery compared to crystalloids [24]. However, there are exceptions; 4% albumin was associated with a 15% larger hemorrhage vs. Ringer´s in a study of 1,386 patients undergoing cardiac surgery [25].

### The oncotic pressure re-distributes fluid

**Fig. 1:**
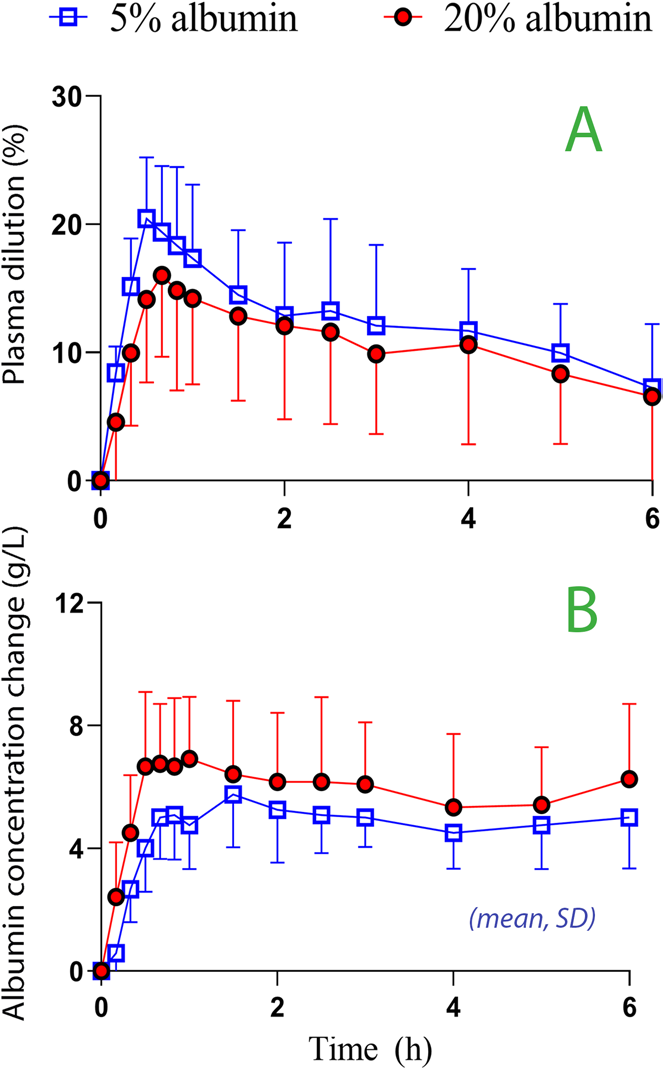
Similarity between albumin 5% and 20% when the same amount of albumin is given intravenously. (**A**) The plasma dilution, which is a surrogate for plasma volume expansion. (**B**) The increase in plasma albumin concentration. Twelve volunteers received 0.6 g of albumin as a 5% and 20% solution in a crossover fashion on different occasions (approximately 200 and 800 mL, respectively). Calculations by mass balance. From reference 26

The oncotic pressure of the plasma is 22–25 mmHg while albumin 5% has a pressure of 19 mmHg and albumin 20% has 160 mmHg [26]. This suggests that some of the volume of the 5% solution would quickly leak into the interstitium while albumin 20% recruits fluid from the interstitium. The transfer is difficult to calculate theoretically because an infusion increases the capillary hydrostatic pressure, which promotes capillary leakage. Moreover, the interstitial albumin concentration increases when albumin 20% recruits fluid, which counteracts the fluid transport. However, kinetic studies have clarified how the oncotic-driven fluid distribution operates in a circuit involving plasma-interstitial-lymph flow [26]. Lamke & Liljedahl used radio-albumin to measure the PVE response to 4%, 5%, and 20% albumin in postoperative patients [27]. They concluded that the PVE is determined by the amount of infused albumin and not by the infused fluid volume. Hence, infusing a surplus of albumin binds more plasma water. Each gram of albumin was claimed to bind 11 mL of water in the plasma. This implies that infusing a pre-determined amount of albumin as a 5% or 20% solution would yield the same PVE and plasma albumin pattern after a brief period of additional expansion due to the crystalloid component of the 5% albumin solution, which quickly leaves the plasma space. This proposed similarity has been confirmed in a cross-over experiment in volunteers [26] (Fig. 1).

Margarson & Soni infused 200 mL of albumin 20% and found that the hemodilution was 1.5 times greater as expected from the infused volume alone in volunteers and 1.15 times greater in septic patients [28]. The finding that the PVE exceeded the infused volume was attributed the oncotic-driven secondary distribution of fluid.

The equilibration of the oncotic pressure between the plasma and interstitium was challenged 15 years ago by advocates for the Revised Starling principle, which is a theory based on laboratory experiments in primitive animal systems. The revised principle holds that high oncotic pressure cannot withdraw fluid via the transcapillary route (“non-absorption rule”) and that plasma, or plasma substitutes, cannot achieve a sustained supranormal plasma volume or reduce tissue edema [29]. The ability of hyper-oncotic albumin to recruit interstitial fluid is still being questioned [30, 31]. However, recent studies show that albumin 20% does indeed recruit interstitial fluid but demonstrated that the transfer primarily occurs via the lymphatic route [32]. Such recruitment operates more slowly than the transcapillary route, delaying the maximum PVE to 10–20 min after an infusion ends, which is an observation already made 20 years ago by Margarson & Soni [28]. Based on hemodilution data, each milliliter of infused albumin 20% recruits 3.4 mL of interstitial fluid of which approximately 2.5 mL is excreted as urine [33]. Hence, the PVE approximately twice as large as the infused volume, which has been demonstrated by several studies from our group and by others [26, 33–40].

### Volume kinetics

**Fig. 2:**
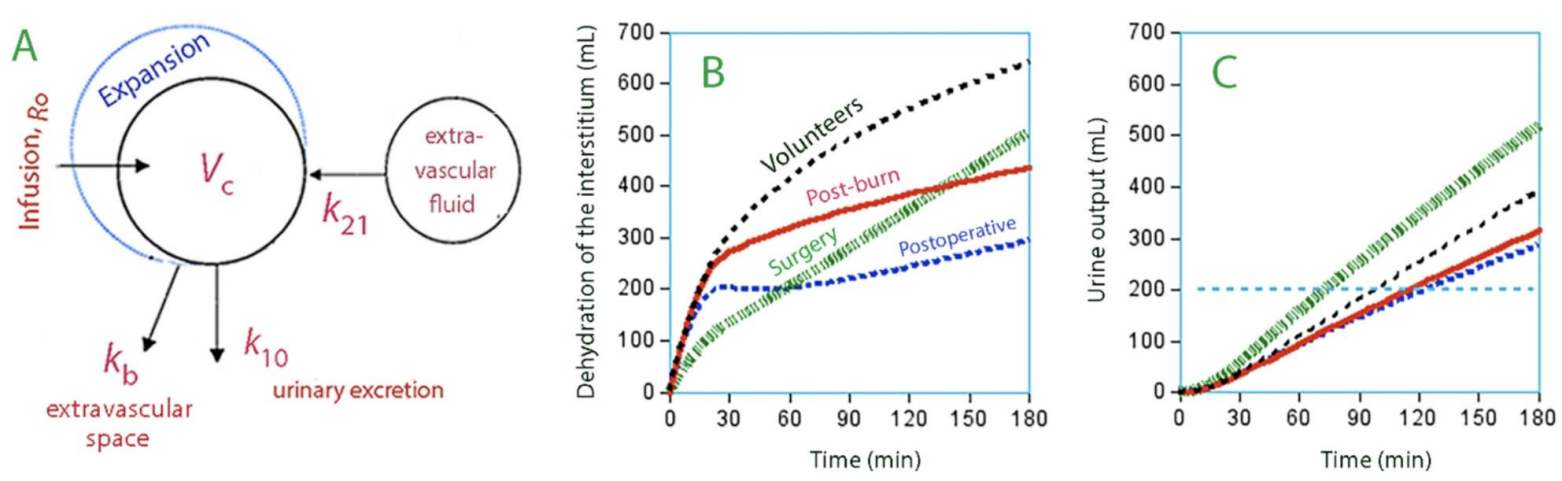
**(A)** The kinetic model used to study the turnover of the fluid volumes when albumin 20% is infused intravenously **(B)** Simulated dehydration of the interstitial space over 3 h when 200 mL of albumin 20% is infused over 30 min. Each curve is based on 15 experiments. The dehydration was taken as the difference between the two flows denoted *k*_21_ and *k*_b_ in subplot A. Based on data taken from references 34, 35, och 40. **(C)** Urine output during the same experiments. The horizontal dotted line marks when the body has lost more volume than was infused

A volume model has been designed for albumin 20% (Fig. 2A) which quantifies the recruitment of interstitial fluid and the half-life of both albumin and fluid [34]. The model has been applied to volunteers [33, 37], post-burn patients [40], to patients during surgery associated with low-grade hemorrhage [35] and on the morning after major cancer surgery [36]. The infusion volume has been 3 mL/kg (approximately 200 mL) over 30 min. The kinetic parameters for these study groups were quite similar, the chief difference being that the body weight was higher and the size of the central volume (*V*_c_) was larger for the post-burn patients, likely reflecting intensive volume loading on admission. Moreover, the rate constant for capillary leakage was greater in two groups (post-burn and postoperatively) likely associated with moderately severe inflammatory response as demonstrated by a mean C-reactive protein concentration of 70–90 µg/L.

An interesting difference from crystalloid fluid is the similar estimates of *k*_10_ for awake and anesthetized subject while, during general anesthesia, *k*_10_ of crystalloid fluid is only 10–15% of what is found in the awake state [41, 42]. Hence, the dependency of arterial pressure for urine output is much smaller for albumin 20% than for crystalloid fluid, which results in that albumin 20% is the better diuretic at low mean arterial pressures (< 70 mmHg) [43]. An infusion time up to 2 h does not matter much to the maximum PVE and the half-life [37].

Simulations based on the kinetic parameters suggest that i.v. infusion of albumin 20% over 30 min recruits the same amount of interstitial fluid, which does not occur when 5% albumin is infused. The effect is attenuated if the infusion is given slowly, but an infusion time of 2 h still provides good recruitment [37]. The interstitial compartment is thus dehydrated, and the process is continued at a lower rate post-infusion (Fig. 2B) because the urine output exceeds the concomitant decrease of the plasma volume (Fig. 2C). The half-life of the infused fluid volume varies between 6 and 10 h except during surgery when 21 h has been found [35].The half-time of the intravascular persistence of the albumin molecules is longer as they are not excreted [44]. The half-life must be kept in mind when prescribing albumin; one fourth of the volume expansion induced on one day remains the next day. Thus, prescribing albumin daily poses a risk of volume overload.

In the clinical setting, the kinetics of albumin 20% is modestly dependent on the plasma albumin concentration when an infusion begins. Low plasma albumin results in a greater relative PVE and a faster leakage rate of fluid [45, 46]. Accelerated urine flow is seen in volunteers and in mixed cohorts [43] but the effect might be overshadowed in the clinic due to other factors that retain fluid. How the capillary leakage rate of albumin is affected depends on the reason for the low plasma albumin level [44]. The influence of an already expanded interstitial space on the kinetics of albumin 20% is unclear, but the oncotic-driven recruitment of interstitial fluid in overhydrated postburn patients is not accelerated (Fig. 2B). A low blood hemoglobin level also increases the capillary leakage rate of albumin 20% [37] but the effect is not unique for this fluid.

### Urine flow

The urine output averages 3 times the infused volume over a 5-h period after a bolus infusion of 200 mL of albumin 20%, and reductions of the urine creatinine level confirms a diuretic effect compared to steady state [26, 35, 37]. The accelerated diuresis is probably due to improved renal perfusion and maintained blood viscosity despite hemodilution. A traditional way to dehydrate patients with edema in the ICU setting is to combine 20% albumin with furosemide, but we recommend allowing the diuretic response of the albumin bolus to occur before administering furosemide due to the intrinsic diuretic properties of the albumin 20%.

A two-stage effect on kidney function can be anticipated. From a physiological point of view, a rise in plasma COP would retain fluid in the glomeruli. However, case reports show that excessive amounts of hyper-oncotic fluid are needed before anuria/oliguria develops [47–49]. Nevertheless, a study of 1,013 patients reported a 6-fold increased risk of anuria when 20% albumin was used to reverse hemodynamic instability in ICU patients [50] but the validity of this clinical study has been debated [51]. Three literature reviews also found no increased incidence of acute kidney injury (AKI) after treatment with albumin 20% compared to crystalloid fluid [51–53].

### Effects on the glycocalyx

Albumin reduces the capillary permeability, and early studies on the hydraulic conductivity (Lp) of capillaries suggested that positive charges on albumin were required for this effect. Chemical modification of arginine residues on the surface of the albumin eliminated the Lp-reducing effect [54]. At the time, it was thus believed that charge-related interactions between albumin’s arginine residues with the negatively-charged glycocalyx are responsible for the reduction of water permeability across the endothelium [54]. Other possibilities included albumin absorption to the walls of the cell junction that, in turn, would reduce water flux [55]. However, Comper [56] challenged the idea that charge-selectivity participates in albumin permeability across the glycocalyx. He showed that the clearance of plasma proteins does not change despite a 3-fold change of the interstitial hydrostatic pressure [57].

Osmotic pressure measurements assessing the interaction of albumin with glycosaminoglycans (GAGs) and other polymers is a sensitive method for measuring ionic interactions. Studies on the interaction of albumin with heparan sulfate and hyaluronan, considered to be major components of the endothelial glycocalyx, demonstrated the interaction coefficient is small and independent on ionic strength [58, 59]. Importantly, there is a lack of correlation of heparan sulfate and GAG content of the endothelial glycocalyx with albumin permeability. Genetic deletion of major heparan sulfate and GAG-synthesizing enzymes had little effect on the development of proteinuria [60–62]. Hence, the effects of albumin on permeability through the glycocalyx appears to result solely from size and volume exclusion.

How do we reconcile the historically alleged effects of albumin’s charge on endothelial permeability given the biophysical data demonstrating that albumins effects on permeability are purely a function of size exclusion and not related to ionic effects? The answer lies in the fact that albumin carries small, biologically active compounds that influence endothelial permeability. For example, albumin shuttles sphingosine-1-phosphate (S1P) between red blood cells and the endothelial cell with subsequent enhancement of barrier function [63]. When the S1P concentrations of red blood cells were reduced below normal, albumin lost its effect on microvascular permeability. Thus, the transport of S1P from the plasma and erythrocytes to the endothelial surface is dependent upon albumin and the subsequent activation of the endothelial S1P receptor, resulting in increased cortical actin [64]. Therefore, the protein-dependent effects long attributed to albumin as a component of the permeability barrier have been revised to its volume excluding effect and transport of S1P [63]. Similarly, albumin-dependent S1P transport to the endothelial surface inhibits metalloproteinases and, thus, protect the glycocalyx from degradation [65].

Albumin is also reported to regulate endothelial cell calcium. When vessels were perfused with an albumin-free solution, intracellular calcium increased; when albumin was added back to the perfusate, intracellular calcium returned to baseline values [66]. The increase in intra-cellular calcium was associated with an increase in permeability. It is unknown whether this effect was caused directly by albumin or a secondary molecule that it was carrying.

In summary, the clinical use of hyper-oncotic albumin will raise plasma albumin concentrations and, therefore, the amount of albumin within the endothelial glycocalyx. However, the effects of albumin on capillary wall permeability are likely due to changes in COP and changes in the void volume of the glycocalyx secondary to size exclusion.

### Antioxidative properties

Free radicals and reactive oxygen species are normal by-products in cellular oxygen metabolism. These contribute to oxidative stress and damage to proteins or DNA, which is linked to numerous diseases. The human organism has developed multiple protective (antioxidant) systems scavenging or converting free radicals such as enzymes (superoxidase dismutase, catalase, peroxidase) and uses minerals like selenium, magnesium or zinc, and vitamins like vitamin A, C, or E [67, 68].

Albumin itself represents an abundant antioxidant [69]. On one side, the antioxidant properties of albumin rely on the binding capacity for ligands such as copper or iron, limiting their participation in the Fenton reaction forming hydroxyl radicals. In addition, albumin offers indirect antioxidative properties by binding and, therefore, protects oxidizable substrates such as polyunsaturated fatty acids or inhibiting lipid peroxidation by binding bilirubin and homocysteine, which itself may act through oxidation of low-density lipoproteins. On the other side, albumin is able to scavenge hydroxyl radicals by its reduced cysteine residue, Cys34 [69]. More than 70% of the free radical-trapping activity of the serum has been attributed to serum albumin using a free radical-induced hemolysis test [70]. The antioxidative capabilities of albumin might be impaired by damages to the structure of the molecules such as increased glycation in diabetic patients, reducing binding capacity for copper and iron, or exerting toxic effects by itself [69, 71].

## Perioperative use of albumin 20%

### Preoperative

Low pre-operative plasma albumin level can be used as a marker to predict poor postoperative outcomes such as delirium, complications after colorectal cancer surgery, or survival after ovarian cancer [72–74]. This association is considered to point at the state of health or underlying disease and is not an indication for administration of exogenous albumin. Despite this view, correcting preoperative hypoalbuminemia prior to off-pump coronary artery bypass surgery improved perioperative urine output and reduced the incidence of postoperative AKI, albeit not severe AKI or need for renal replacement therapy [75]. However, the comparator was sodium chloride, which might induce AKI by itself [76]. Therefore, indications for the preoperative use of albumin remains to be elucidated. Albumin has also been used for acute preoperative hemodilution in older studies, a procedure not in use anymore [77, 78].

### Intraoperative

Löffel et al. quantified the blood volume expanding property of albumin 20% during cystectomy and found it to average 1.9 mL per infused mL over 5 h [38]. The method of study was mass balance followed by a regression analysis that took both the amount of infused Ringer and the surgical hemorrhage into account.

A further study compared the ability of albumin 20%, albumin 5%, and Ringer to maintain the blood volume during cystectomies associated with an average blood loss of 848 mL [39]. The analysis showed that 3 mL/kg of albumin 20% given together with 1 mL/min of Ringer maintained the blood volume over a 5-h period of surgery. Providing the same amount of albumin as a 5% solution resulted in mild hypervolemia (+ 100 mL) while Ringer given in the 3:1 proportion to the bleeding was associated with a diminution of the blood volume (-313 mL).

**Fig. 3:**
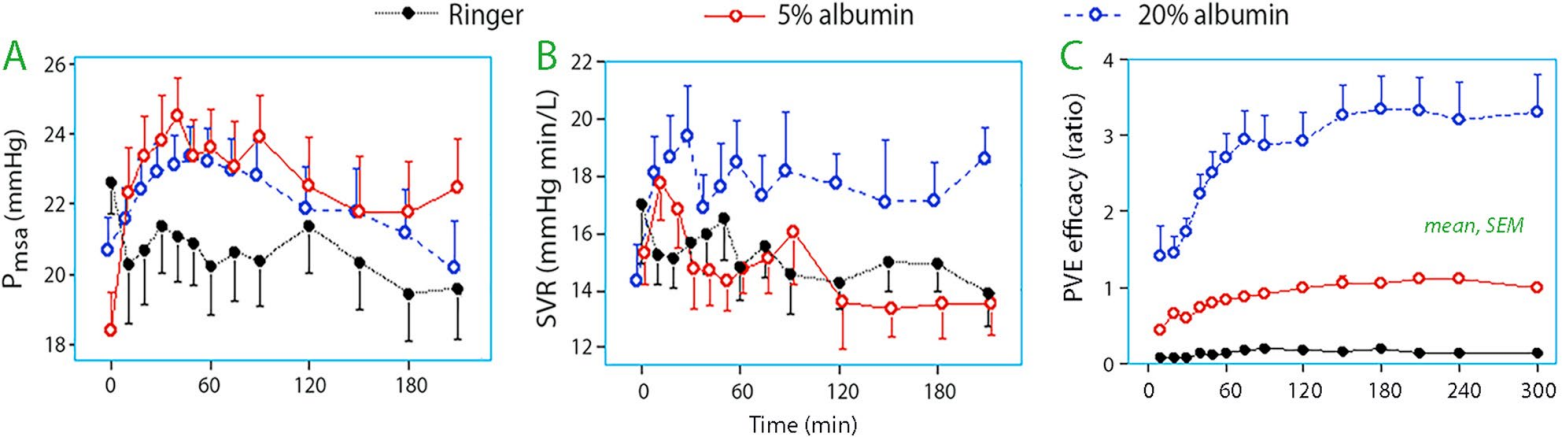
**(A)** Mean systemic filling pressure (P_msa_), **(B)** systemic vascular resistance (SVR), and the plasma volume expansion (PCE) efficacy of Ringer´s lactate, 5% albumin and 20% albumin when infused during major surgery associated with hemorrhage that averaged 848 mL. The PVE efficacy is obtained as [(blood volume change + blood loss) / infused volume]. Based on data taken from reference 39

A secondary analysis showed that the mean circulatory filling pressure increased with albumin but not with Ringer (Fig. 3A). A hemodynamic detail is that albumin 20% increased the systemic vascular resistance when infused during surgery [79] (Fig. 3B) which is probably due to its high viscosity. This quality is believed to be beneficial to the blood rheology and the microcirculation [80, 81], which has recently been confirmed in sepsis, although improvement was limited [82].

The PVE efficacy of the albumin solutions during major surgery hardly becomes attenuated over time (Fig. 3C**).** This variable can be obtained as [(blood volume change + blood loss) / infused volume]. In the extended study in which cystectomy patients were randomized between three fluid programs, PVE showed efficacies of 0.7 for 5% albumin, 2.1 for 20% albumin, and 0.2 for Ringer [39]. In volunteers this variable is 0.7% for albumin 5% and approximately 2.0 for albumin 20% [26, 33, 83]. As mentioned, the same calculation during major surgery yielded 1.9 for 20% albumin [38].

**Fig. 4:**
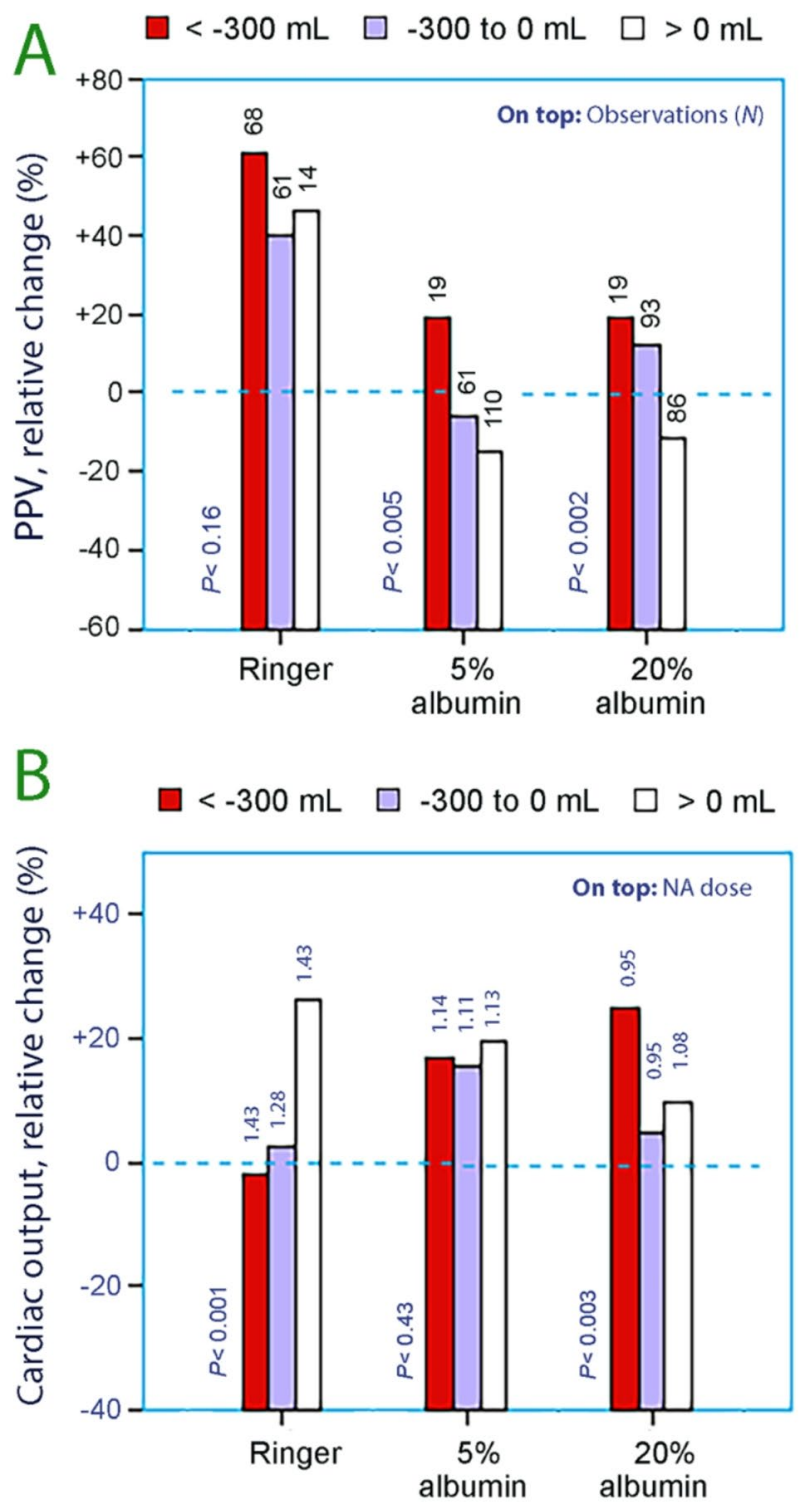
**(A)** The relative change in pulse pressure variation (PPV) from baseline for different degrees of hypovolemia depending on which infusion fluid was used to combat hemorrhage during major surgery. The number of observations is given on top of each bar. **(B)** Indication of hypovolemia by using the relative change in cardiac output, which is probably well maintained due to influence of surgical stress and the use of norepinephrine (NA). The mean dose of NA in mg is given on top of each bar. Each fluid groups consisted of 14 patients. Based on data taken from reference 79

The ability of hemodynamic measures to detect hypovolemia was also affected by using albumin as infusion fluid. PPV works well with the albumin solutions while cardiac output appears to be suitable when Ringer is used (Fig. 4) [79]. The different viscosities of the fluids probably play a role for these differences.

Gunnström et al. used the same infusion regimen consisting of albumin 20% (3 mL/kg over 30 min) in 15 patients undergoing lengthy surgery with minimal hemorrhage (200 mL) [35]. The complete volume kinetic analysis yielded parameters values close to those obtained in volunteers, except that the oncotic-driven interstitial fluid recruitment was weaker (Fig. 2B**)**. In that study, norepinephrine was infused in 42% of the studied time periods; it was demonstrated to decrease the PVE and increase the urine flow in a dose-dependent fashion.

Despite these positive effects of 20% albumin and reductions of tissue edema, overall outcomes are not changed. This suggests that inflammatory, immunological, and infectious complications dominate postoperative outcomes. However, Vlasov et al. found less myocardial damage using 4% albumin instead of Ringer during and after cardiac surgery, but the infused volumes then differed [84].

A recent meta-analysis did not show an impact of perioperative use of albumin on postoperative renal function or mortality despite lower overall fluid balance [85].

Preemptive administration of 20% albumin during pancreatectomy did not reduce the number of postoperative complications as measured by the Comprehensive Complications Index despite lower intraoperative fluid balance [86].

In cardiac by-pass surgery, intraoperative use of 20% albumin decreased the incidence of pleural effusions [87]. By contrast, the use of 4% albumin did not reduce pulmonary edema [88], atrial fibrillation [89], or major adverse events [90].

### Postoperative

Postoperative low albumin levels are associated with impaired outcomes after cardiac and non-cardiac surgery [91–93], just as it is with preoperative hypoalbuminemia.

Hyper-oncotic albumin reduces the need for crystalloid fluid boluses after cardiac surgery, which increases the time before vasopressors are needed when compared to crystalloid resuscitation [94]. Nevertheless, a study of postoperative patients in shock showed a 10% higher incidence of AKI when patients had been exposed to hyper-oncotic albumin [95]. However, in that study approximately half the patients received hetastarch, and the dose of administered crystalloids (0.9% saline or Ringer) was not quantified [95].

In a retrospective study, the supplementation of albumin did not improve wound healing after total knee arthroplasty [96]. In addition, maintaining serum albumin levels above 30 g/L in high-risk surgical patients before discharge from the postoperative care unit, did not reduce postoperative complications when using the Clavien-Dindo Classification ≥ 2 [97].

Volume resuscitation using albumin seems not to impair coagulation after cardiopulmonary bypass to the same extent as gelatin or hydroxyethyl starch [98, 99].

## Albumin 20% in intensive care

Critical illness impacts the synthesis and degradation rates of albumin and also modifies the lymphatic flow, resulting in hypoalbuminemia and altered distribution [89]. In these situations, associated with lower COP, the administration of hyper-oncotic albumin clearly increases the intravascular COP and might even re-establish the normal transcapillary COP gradient. Moreover, positive fluid balance is common in the ICU setting and leads to poor outcomes, and 20% albumin offers a strong PVE effect while keeping the volume of administered fluid low. Dubois et al. reported improved organ function score by administrating albumin 20% to 100 severely and modestly hypoalbuminemic ICU patients [100]. This approach could have inspired others to design clinical studies of albumin, such as ALBIOS and SAFE, to restore a low plasma albumin concentration [21, 101]. However, this is not an accepted indication for albumin administration today except for very special diagnoses that are beyond the scope of this review.

Mårtenson et al. reported, in the framework of the SWIPE Trial, that using albumin 20% decreased the overall resuscitation fluid volumes compared with albumin 5%, thus reducing the early positive fluid balance without being associated with harm; the mean difference was − 576 mL (95% CI -1030 to -120 mL; *p* = 0.01) after 48 h of intervention [15]. The survival after ICU was slightly but significantly better in the groups treated with albumin 20% (97% vs. 91%; *p* = 0.02).

Hyper-oncotic albumin may have a role during de-escalation (i.e. removing excessive fluid) after hemodynamic based fluid optimization in septic patients. Removing excessive fluid is usually achieved by spontaneous diuresis [102], but a benefit of using 20% albumin during de-escalation has been found in lung injury. Cordemans et al. showed, in a retrospective study, that small volume resuscitation with 20% albumin combined with furosemide or ultrafiltration for fluid removal reduced in positive fluid balance, extravascular lung water index, and intraabdominal pressure [103]. This is probably a benefit because administration of diuretics favors restoration of the microcirculation and improves oxygen extraction rate after treatment in the ICU [104]. Furthermore, Martin et al., could show in 40 patients with acute lung injury/acute respiratory distress syndrome that infusing 100 mL of 20% albumin every 8 h for 3 days in combination with furosemide reduced the positive fluid balance almost a factor of four resulting in better oxygenation index [105]. These reports are in line with a review article by Haynes et al. which reported that albumin (any concentration) decreases pulmonary edema and respiratory dysfunction compared with crystalloid in acute illness [106].

Infusion of albumin during surgery clearly creates a PVE that is superior to crystalloids for many hours [39] but the efficacy of iso-oncotic colloids in some ICU studies has been poorer than the expected 4:1 ratio [107, 108]. These comparisons have been based on physiological endpoints, extended over long time periods, and used preparations with intravascular half-lives being shorter than albumin; both 6% hydroxyethyl starch 130/0.4 and 4% succinylated gelatin have a half-life of 2 h [109, 110]). Kinetic simulations support that the PVE would be the same for hydroxyethyl starch and Ringer 24 h after an infusion [111] although equivalence would be reached later for albumin. Poor diuretic response to volume expansion during general anesthesia and stressful settings helps to maintain a prolonged low-level PVE when crystalloid is used [43].

### Sepsis

The most severe degrees of hypoalbuminemia are seen in septic patients. It is usually attributed to increased capillary permeability due to glycocalyx injury [112] although inhibited lymphatic flow is also a contributing factor to both hypoalbuminemia and peripheral edema [113, 114]. Infusion fluids are also maldistributed during sepsis due to release of cytokines and nitrous oxide, which decrease the interstitial hydrostatic pressure (“suction effect”), dilate vascular beds, and increase cardiac output [115–118]. The degree of hypoalbuminemia and positive fluid balance are linked statistically [119] and might serve as an indicator of the degree of fluid maldistribution.

The role of albumin for fluid therapy in sepsis has been lively debated [120–122]. Albumin 20% reduces peripheral edema by rectifying the COP gradient, but there is concern that the edema might be aggravated later on due to increased capillary leakage. Margarson & Soni measured the capillary leakage rate of albumin in septic patients and reported it to be approximately 30% higher than expected [123]. Castro et al. found a similar difference when comparing patients with sepsis and COVID-19 [124]. Increased leakage of plasma is normally compensated by accelerated lymphatic flow (“interstitial washdown” [125]) but this does not seem to occur in septic patients.

In addition to the strong PVE and COP effects of albumin, there is hope that protective functions of albumin through oncotic, antioxidant, and anti-inflammatory mechanisms will be helpful to septic patients. However, in the ALBIOS Trial daily infusions albumin 20% in addition to crystalloids, as compared with crystalloids alone, did not improve the survival at 28 and 90 days in non-selected septic patients (44 vs. 50%) [21]. No significant difference in the incidence of AKI between the groups was found, but survival was improved in a subgroup of patients with septic shock. The opinion at the time of the ALBIOS Trial (2014) was that albumin does not cause harm but there might be an advantage in septic shock [126].

Benefits of albumin administration are reported for other sub-groups with sepsis. A study comparing two consecutive periods (1997–2004 and 2005–2010) during which cirrhotic patients with septic shock showed increased survival after implementation of adjuvant treatments including albumin infusion as fluid volume therapy, low-dose glucocorticoids, and intensive insulin therapy [127]. During the first 7 days, patients in the albumin group, as compared with those in the crystalloid group, had a higher mean arterial pressure and less positive fluid balance. Moreover, a recent trial investigating the effect of 5% albumin versus saline 0.9% in 154 cirrhotic patients with sepsis related hypotension showed improved hemodynamic stabilization and 7-day survival in the albumin group [128].

A subgroup analysis of the SAFE study indicated that albumin 4% reduces the mortality in septic patients [99, 129]. Pooling the existing clinical trials of albumin in septic patients up to 2014 [130] or the hyper-oncotic albumin up to 2024 [131] yield a statistically significant lower mortality for albumin in septic shock by approximately 20%. Therefore, studies are currently ongoing to finally demonstrate benefit of albumin in this condition.

Another meta-analysis of albumin in sepsis by Geng et al. showed that patients who received albumin 4% and 5% had a better outcome than those treated with crystalloid fluid (odds ratio 0.85) but the best effect was achieved by 20% albumin (odds ratio, 0.81 [122]. By contrast, the RASP study, which compared albumin 4% versus crystalloid alone during the first 6 h after ICU admission in 360 cancer patients with severe sepsis or septic shock, confirmed no difference in 7-day or 28-day survival [132].

The very recent ARISS study (2026) found that the early administration of 20% albumin targeting a plasma concentration of 30 g/L in patients with septic shock did not significantly reduce the 28-day mortality (31% vs. 39% in controls, primary outcome) or the 90-day mortality (43% vs. 46%). There was no detectable effect on organ failure or fluid balance. The premature termination due to poor recruitment necessitates cautious interpretation, though the intervention demonstrated a safety profile similar to standard care [133].

### Microcirculation

Capillary flow is maldistributed in sepsis due to shut down of flow in some areas while shunting occurs in others [134]. Albumin improves the microcirculation in septic patients as obtained by a side-stream darkfield analysis, although the effect is not dramatic [82]. Infusion of albumin 4% or 20% in a rat model of normotensive endotoxemia produced a similar acute improvement of the microvascular perfusion as to become aligned with unresuscitated animals [135].

Gabarre et al. studied 50 patients with sepsis or septic shock and found that the capillary refill time (< 3 s) was more frequently normalized in patients receiving albumin compared to those given saline (63 vs. 29%, *p* = 0.02) [136]. Such restoration did not correlate with cardiac output variations. Furthermore, albumin but not saline was further associated with decreasing plasma lactate concentrations. According to Lara et al., septic patients with pathological capillary refill time haver a poorer prognosis compared to septic patients without this sign [137].

There is a hope that the pleiotropic physiological effects of albumin, such as antioxidative and anti-inflammatory properties, would protect and stabilize the glycocalyx and the endothelium, with positive effects on vessel wall integrity and a reduction of the capillary leak [138]. Hariri et al.. studied 35 septic patients and found that a bolus of 100 mL of albumin 20% over 15 min had a beneficial effect on the endothelial function as assessed by the acetylcholine iontophoresis [139]. Infusing 20% albumin into hypoalbuminemic septic patients does not increase or decrease the capillary leakage of albumin [123]. However, the influence of the choice of infusion fluid on endothelial function during sepsis is a quite active research field, but the precise mechanism and circumstance during which albumin would be helpful is unknown [140].

### Late edema

The half-life of exogenous albumin in the body is considerably longer than the intravascular half-life. It leaks from the plasma to the interstitial space and returns to the plasma as part of the lymphatic flow. Hence, infused albumin spends the remainder of its life cycle flowing in a circuit between the plasma and the interstitial space. The endogenous synthesis is reduced when exogenous albumin is given, whereby the risk of hyperalbuminemia is reduced.

Late edema is still a possible adverse effect because the albumin maintains its oncotic pressure also after having entered the interstitial space. In a healthy person, increased capillary leakage of fluid and albumin accelerates the lymphatic flow within minutes [141] and equilibrium is achieved within 30 min [113]. Our opinion is that albumin administration may primarily aggravate peripheral edema in the earlier stages of conditions with maldistribution of fluid, of which slow lymphatic pumping is a typical feature [114, 118, 142, 143]. General anesthesia creates a temporary mild maldistribution of fluid [108] while more severe forms occur in acute inflammatory diseases, such as sepsis and burns [109, 137]. When the condition has stabilized the risk is probably small and should be weighed against the therapeutic benefits of using albumin 20%, i.e., rapid acute redistribution of fluid in favor of the plasma volume and accelerated urine flow.

## Differences between albumin 5% and 20%

The main difference between albumin 5% and 20% is that the hyper-oncotic preparation dehydrates the interstitium (Fig. 2B). Lymph rich in immunoglobulins and other proteins is recruited, which limits effects of fluid-induced hemodilution on their plasma concentrations [32]. Maximum PVE occurs with a delay of 10–20 min after an infusion ends (Table 1).

**Table 1:** Key summary points for 20% albumin and comparison of 20% Albumin vs. 5% albumin and crystalloids

Feature	20% Albumin	5% Albumin	Ringer
Expansion power	Twice the infused volume	~ 70–100% of infused volume	~ 20–30% of infused volume
Expansion half-life	6–10 h (typically 6.5–7.6 h)	~ 2.5–5 h	Short (~ 20–40 min)
Primary driver	Determined by the amount of infused albumin, not by the fluid concentration	First: Infused volume. Later: infused amoumt of albumin	Infused volume
Fluid recruitment	Recruits from interstitium via lymphatics	Negligible recruitment; maintains volume	Leaks *into* interstitium (edema)
Cardiac response	Strong/sustained response	Modest response	Brief response; dissipates fast
Diuretic effect	Sustained at low MAP (< 70 mmHg)	Modestly MAP-dependent	Rapidly lost at low MAP
Hydration status	Net dehydrating (interstitium & body)	Neutral to positive fluid balance	Promotes positive fluid balance
Kinetic stability	Similar in healthy vs. intra- or postoperative states	Variable in sepsis/leak	Highly variable

• **Recruitment Efficiency**: For every 1 mL of 20% albumin infused, it recruits roughly 3–4 mL of fluid into the blood

• **Clinical Advantage**: It allows for “small-volume resuscitation,” achieving hemodynamic targets with roughly “one-third” the volume required by 5% solutions

The iso-oncotic 5% solution also has a mild dehydrating effect on the interstitium, but this appears with a delay of 4–5 h [26]. The maximum PVE occurs with a delay of 0–5 min after an infusion ends.

The urine output exceeds the infused volume 1–2 h after a 30-min infusion of albumin 20% (Fig. 2C) while this point is delayed > 6 h for albumin 5%.

In volunteers, the PVE is somewhat greater (+ 20%) and plasma albumin lower (-20%) for albumin 5% than for albumin 20% when the same amount of albumin is administered, but most of the difference cancels out over a 2-h period [26, 39] (Fig. 1). The lower plasma albumin level with albumin 5% probably results in a faster capillary leakage rate of fluid.

Albumin 5% is more effective than albumin 20% to increase the mean systemic filling pressure during surgery (Fig. 3A). By contrast, only albumin 20% increases the vascular resistance, which consequences have not yet been debated (Fig. 3B).

## Recommendations and conclusions

The conclusions given below are based on our readings and own studies of albumin 20% conducted during the past decade. Hyper-oncotic albumin is currently not the first-line fluid choice in surgery or intensive care and it does not have strong support in dehydrated or hemodynamically unstable patients, and in the most acute phase of acute inflammatory conditions. However, it may benefit patients in special situations that are supported by evidence. These include:

1. Surgery requiring large amounts crystalloid fluid (Goal: to avoid adverse effects due to fluid overload).
2. During the de-escalation phase in ICU patients (Goal: dehydration without causing hypovolemia, improve the microcirculation).
3. To expand the plasma volume in patients with edema, with particular benefit in those with low urine output (Goal: re-distribute interstitial fluid to the plasma volume).
4. Gastrointestinal surgery with suturing in patients requiring large amounts of crystalloid fluid and/or have developed sepsis after such surgery (Goal: decrease the risk of suture insufficiency).
5. Lung injury, pleural effusions, respiratory distress syndrome (Goal: withdrawing fluid from lung tissue).
6. Septic shock. (Goal: potentially better survival).

## Data Availability

No datasets were generated or analyzed during the current study.

## Abbreviations

AKI: Acute kidney injury
COP: Colloid osmotic pressure
GAG: Glycosaminoglycan
ICU: Intensive care unit; PVI: plasma volume expansion; S1P: sphingosine-1-phosphate
RASP study: Lactate Ringer versus Albumin in Albumin Sepsis therapy
ROS: Reactive oxygen species
SAFE study: Saline versus Albumin Fluid Evaluation study
